# Reduced expression of DACT2 promotes hepatocellular carcinoma progression: involvement of methylation-mediated gene silencing

**DOI:** 10.1186/1477-7819-11-57

**Published:** 2013-03-07

**Authors:** Sheng Gao, Zhe Yang, Zhi-Yun Zheng, Jia Yao, Feng Zhang, Li-Ming Wu, Hai-Yang Xie, Lin zhou, Shu-Sen Zheng

**Affiliations:** 1 Key Laboratory of Combined Multi-organ Transplantation, Ministry of Public Health, Hangzhou, China; 2 Key Laboratory of Organ Transplantation, Hangzhou, Zhejiang Province, China; 3 Division of Hepatobiliary and Pancreatic Surgery, Department of Surgery, First Affiliated Hospital, Zhejiang University School of Medicine, Hangzhou, China; 4 Key Laboratory of Combined Multi-organ Transplantation, Ministry of Public Health, the First Affiliated Hospital, Zhejiang University School of Medicine, 79 Qingchun Road, Hangzhou, 310003, People’s Republic of China

**Keywords:** DACT2, Hepatocellular carcinoma, Methylation, Progression

## Abstract

**Background:**

Hepatocellular carcinoma (HCC) is one of the most aggressive malignancies in humans, and its prognosis is generally poor even after surgery. Many advances have been made to understand the pathogenesis of HCC; however, the molecular mechanisms that lead to hepatocarcinogenesis and progression are still not clearly understood.

**Methods:**

The expression of DACT2 in specimens from 30 paired HCCs and an additional 61 HCC patients after liver transplantation was evaluated by quantitative RT-PCR and immunohistochemical analysis. We investigated the methylation status of the DACT2 promoter region. We also analyzed the alterations of the cell cycle, migration and invasion after DACT2 knockdown.

**Results:**

The expression level of DACT2 was significantly lower in HCC tissues than in non-cancerous tissues. Reduced DACT2 expression was associated with large tumor size. DACT2 transcripts were at low levels in hypermethylated liver cancer cells and were restored by exposure to a demethylating agent. Reduced expression of DACT2 in MHCC97L cells induced G1/S arrest, increased cell proliferation, and promoted cell invasion.

**Conclusions:**

Our study suggests that DACT2 is silenced by promoter hypermethylation, and reduced DACT2 can promote liver cancer progression. DACT2 may serve as a novel tumor suppressor gene in HCC.

## Background

Primary liver cancer, predominantly consisting of hepatocellular carcinoma (HCC), is the fifth most common cancer and the third most common cause of cancer-related deaths worldwide. It is estimated that approximately 626,000 new cases of HCC occur annually and nearly as many deaths [1,2]. Cases in China account for more than 50% of the world’s cases because of the high prevalence of chronic hepatitis B virus infection and liver cirrhosis [3,4]. HCC is one of the most aggressive malignancies in humans, and its prognosis is generally poor even after surgery [5-7]. Many advances have been made to understand the pathogenesis of HCC; however, the molecular mechanisms leading to hepatocarcinogenesis and progression are still not clearly understood. Therefore, we are still lacking effective therapies of HCC. For better diagnosis and treatment of HCC, it is crucial to identify the genes responsible for the initiation, promotion, and progression of the disease. A detailed understanding of the molecular mechanisms associated with HCC ultimately could improve our current treatment modalities of this disease. HCC is a highly aggressive cancer with activation of multiple signal transduction pathways and various gene alterations [8-10]. The Wnt signaling pathway has been shown to be involved in cell proliferation, apoptosis, differentiation and motility [11]. This pathway is deregulated in a number of cancers, including HCC [12]. Dapper, antagonist of beta-catenin, homolog 2 (DACT2), located on chromosome 6q27, belongs to the DACT (Dpr/Frodo) gene family. This family consists of DATC1, 2 and 3 [13,14]. Members of the DACT (Dpr/Frodo) gene family have been shown to modulate Wnt/β-catenin signaling by interacting with Dishevelled (Dvl), a central component of Wnt signaling [15-17]. A number of studies have revealed the biological properties of DACT proteins in human cancer. For example, Yau et al. demonstrated that the expression of HDPR1, the human homolog of Dpr, was frequently and significantly downregulated in HCC. Downregulation of HDPR1 frequently occurs through hypermethylation of the promoter region and allelic loss of the locus [18]. Jiang et al. identified DACT3 as an important negative regulator of Wnt/β-catenin signaling and functions as a potential tumor suppressor in colorectal cancer. The expression of DACT3 is consistently suppressed in colon cancer, and its expression is silenced by bivalent histone modification [19]. DACT2 has been described and studied in both fish and mammals [14,20,21]. Although there have been relatively few reports about DACT2 in cancer research, the expression level of DACT2 appears to be reduced in some colorectal tumors. To our knowledge, there have been no studies investigating a possible correlation among the expression of DACT2, the clinicopathological factors in HCC, and the relevance of DACT2 expression to oncogenesis.

In this study, we explored DACT2 gene expression in HCC and its possible association with clinicopathological factors. In addition, the epigenetic regulation of DACT2 in HCC cells was also analyzed.

## Methods

### Study population and tissue samples

Sixty-one HCC patients who had undergone liver transplantation during 2003 and 2005 at our hospital (First Affiliated Hospital, Zhejiang University School of Medicine, Zhejiang, China) were enrolled in this study. The study was approved by the local ethics committee, and informed consent was obtained from all of the patients. Of these patients, HCC was diagnosed either before or after transplantation, which was confirmed by histopathological examination. Complete clinical and laboratory data were available before operation and during follow-up, and all patients were HBV-positive (HBsAg^+^). Distribution of clinicopathological data in the study cohort is given in Table 1. An additional 30 patients with HCC were also included in the present study. Specimens of cancer tissues and adjacent noncancerous tissues were collected from these patients after obtaining informed consent.

**Table 1:** Characteristics of the 61 patients and relationship of DACT2 expression with clinicopathological parameters in HCC

**Variables**	**DACT2 expression**		
	**Low (*n *= 31)**	**High (*n *= 30)**	*P *value*
Age (years)			
≤50	17	18	0.797
>50	14	12	
Gender			
Female	1	4	0.195
Male	30	26	
PVTT			
Negative	21	19	0.791
Positive	10	11	
Preoperative AFP level (ng/ml)			
≤400	15	18	0.444
>400	16	12	
Histopathologic grading			
Well + moderate	24	19	0.270
Poor	7	11	
Tumor size (cm)			
≤5	9	17	0.040
>5	22	13	
Tumor number			
Single	10	10	1.000
Multiple	21	20	

*PVTT*, portal vein tumor thrombi; *AFP*, alpha-fetoprotein.

*Chi-square test (Fisher’s exact test).

### Reverse-transcription PCR and real-time reverse-transcription PCR

Total RNA was extracted from hepatic specimens or cell lines, and cDNA was synthesized from total RNA. The messenger RNA (mRNA) expression level of theDACT2 was determined by RT-PCR. The polymerase chain reaction (PCR) primer sequences for the DACT2 mRNA amplification have been described previously [19]. Real-time PCR reactions were performed by the ABI7500 system (Applied Biosystems, CA, USA) and SYBR green dye (Applied Biosystems).

### Immunohistochemistry

Immunohistochemical studies of DACT2 were performed on surgical specimens from HCC patients with cancerous and noncancerous adjacent tissues. Primary rabbit polyclonal antibodies against DACT2 (ProSci Inc., CA, USA) were used at a dilution of 1:600. Immunohistochemistry was performed as described previously [22].

### Cell lines

Liver cancer cell lines SMMC-7721, HepG2 and Hep3B as well as the metastasis-capable human HCC cell lines MHCC97L (Shanghai Institute of Cell Biology, Shanghai, China) and HCCLM3 (Liver Cancer Institute of Fudan University, Shanghai, China) were purchased. All cell lines were maintained in the recommended culture condition and incubated at 37°C in a humidified environment containing 5% CO_2_.

### Bisulfite modification of DNA

Genomic DNA was extracted from cell lines using the QIAamp DNA Mini Kit (Qiagen, Hilden, Germany). DNA samples were modified using EZ DNA Methylation Golden Kit (Zymo Research, CA, USA). Bisulfite converts unmethylated CpG sites to UpG without modifying methylated sites.

### Demethylation with the DNA demethylating agent 5-Aza-2^′^-deoxycytidine

Liver cancer cells were plated at a density of 5 × 10^4^ cells/ml. After 24 h, cells were treated with 10 μmol/l of the DNA demethylating agent 5-aza-2^′^deoxycytidine (5-aza-dC) (Sigma-Aldrich, St. Louis, MO, USA) for 96 h. DNA and RNA were then extracted from cells.

### MSP and bisulfite genomic sequencing

The methylation status of the DACT2 CpG islands was determined by MSP. The primers for detection of the methylated and unmethylated DACT2 CpG islands were described in the previous study [19]. For bisulfite genomic sequencing, 2 μl of bisulfite-treated DNA was amplified using primers [19]. The PCR products were cloned into a PUCm-T vector. Three to five colonies were randomly chosen and sequenced for plasmid DNA extraction with QIAprep Spin Miniprep Kit (Qiagen, Hilden, Germany).

### RNA interference

Short interfering RNA (siRNA) duplexes (GenePharma, Shanghai, China) were used to downregulate DACT2 expression, and a nonsilencing siRNA was used as negative control. After growth in the plate for 24 h, cells were transfected with 33 nM siRNA using Lipofectamine 2000 transfection reagent (Invitrogen, CA, USA) according to the manufacturer’s instructions. The efficacy of transfection was checked by real-time PCR after 48 h.

### Cell cycle analysis

MHCC97L cells were transfected with DACT2 siRNA or a negative control. After 48 h, cells were harvested and stained with a DNA PREP kit (Beckman Coulter, Fullerton, CA). The percentage of cells in G0/G1, S, and G2/M phase was quantified using flow cytometry analysis according to the manufacturer’s instructions (Cytomics FC 500, Beckman Coulter). Analysis of cell cycle data was performed with Multicycle (Los Angeles, CA) analysis software. All experiments were completed in triplicate.

### Cell invasion and motility assay

Cell invasion analysis was performed using 24-well transwell plates (Millipore, Billerica, MA). Forty-eight hours after RNA interference, the filters were coated with Matrigel (BD Bioscience, San Jose, CA) in the upper compartment and seeded with 200 μl of 0.8 × 10^5^ cells. The lower compartment was filled with cell culture medium supplemented with 15% fetal bovine serum. After 48 h, migrated cells on the bottom surface were fixed with methanol and stained with 0.1% Crystal Violet. The cell motility assay was performed similarly, except the cells were applied into the uncoated filter and incubated for 24 h. The invading cells were examined, counted, and photographed using digital microscopy. Three fields were counted per filter in each group.

### Statistical analysis

The relationship between DACT2 expression and clinicopathological variables were assessed by the chi-square test. The detailed statistical tests used in the study are shown in Results and in the table and figure legends. *P* < 0.05 was considered statistically significant.

## Results

### Analysis of DACT2 expression in clinical samples

**Figure 1:**
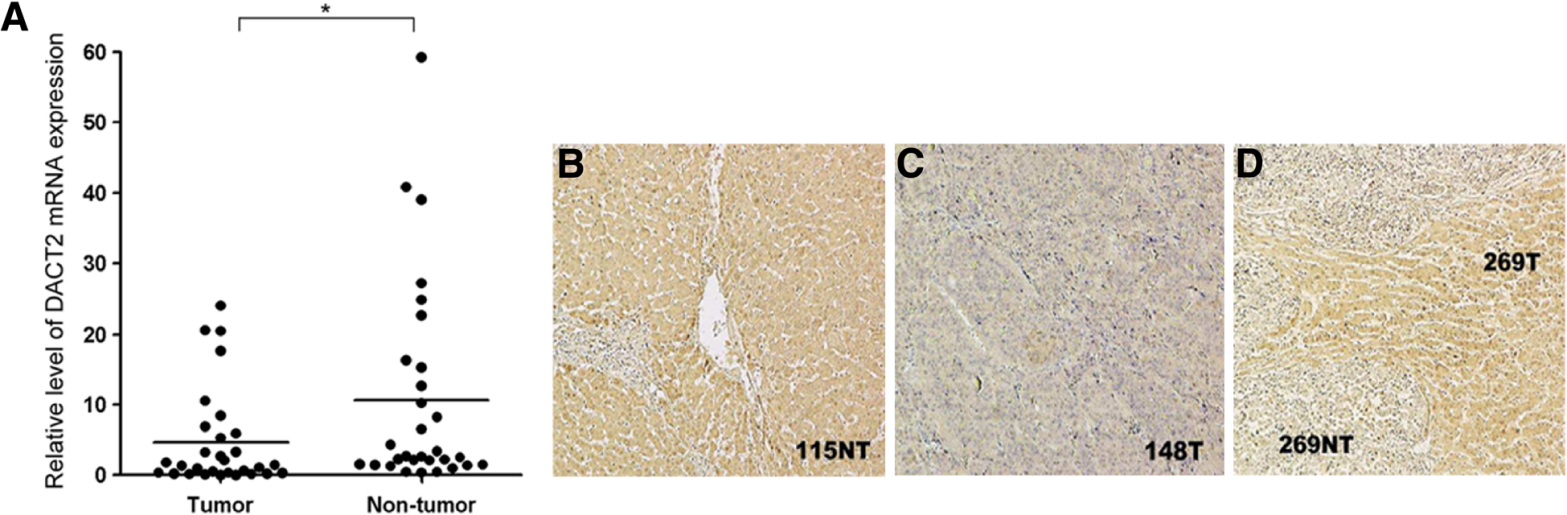
**(A) DACT2 mRNA expression in 30 pairs of clinical samples.** The expression level of DACT2 mRNA was quantitatively determined by RT-PCR for tumor tissue specimens and its corresponding nontumor tissue specimens. The mean expression level of DACT2 in each group was indicated (*P < 0.05, Student’s *t-*test). Representative immunohistochemical staining of DACT2 protein in liver cirrhosis tissue (**B**) and hepatocellular carcinoma section (**C**) and low expression of DACT2 in tumor at the tumor-normal interface (**D**) (original magnification, ×10).

Expression of DACT2 mRNA in HCC tumor specimens was lower than in nontumoral liver specimens in 24 of the 30 paired cases. The average level of DACT2 mRNA expression in HCC tissues was 2.25-fold lower than in adjacent noncancerous tissues (4.68 ± 6.96 vs. 10.55 ± 14.6; *P* < 0.05) as shown in Figure 1A. DACT2 protein expression was also investigated by immunohistochemistry in tumor tissues and the nontumoral liver counterparts (*n* = 26), as is shown in Figure 1B,C,D. In healthy liver tissues, DACT2 was almost homogeneously expressed in non-neoplastic hepatocytes. In tumor tissues, two staining patterns could be distinguished. In most patients (21/26 cases), DACT2 expression was markedly decreased in tumor tissues compared with nontumorous liver tissues. Only five samples showed comparable expression levels of DACT2 between tumor and noncancerous liver specimens.

### Correlation of DACT2 expression with clinicopathological parameters

In this study, we also included another cohort of 61 HCC patients who had undergone liver transplantation. We divided them into two expression groups. The low-expression group (30 samples) included patients whose tumor tissue expressed DACT2 mRNA below the median level. The high-expression group (*n* = 31) consisted of patients whose tumor tissue expressed DACT2 mRNA above the median value. Clinicopathological parameters are listed in Table 1 in relation to DACT2 mRNA expression status. In the relationship between DACT2 expression and the clinicopathological data, we found that DACT2 expression was negatively correlated with tumor size (*P = 0.04*). Tumors with low DACT2 expression were significantly larger in size (>5 cm) than those with high expression. There was no statistically significant correlation between DACT2 expression and age, gender, AFP, histopathological grading, or tumor number.

### Downregulation and promoter methylation of DACT2 in liver cancer cells

**Figure 2:**
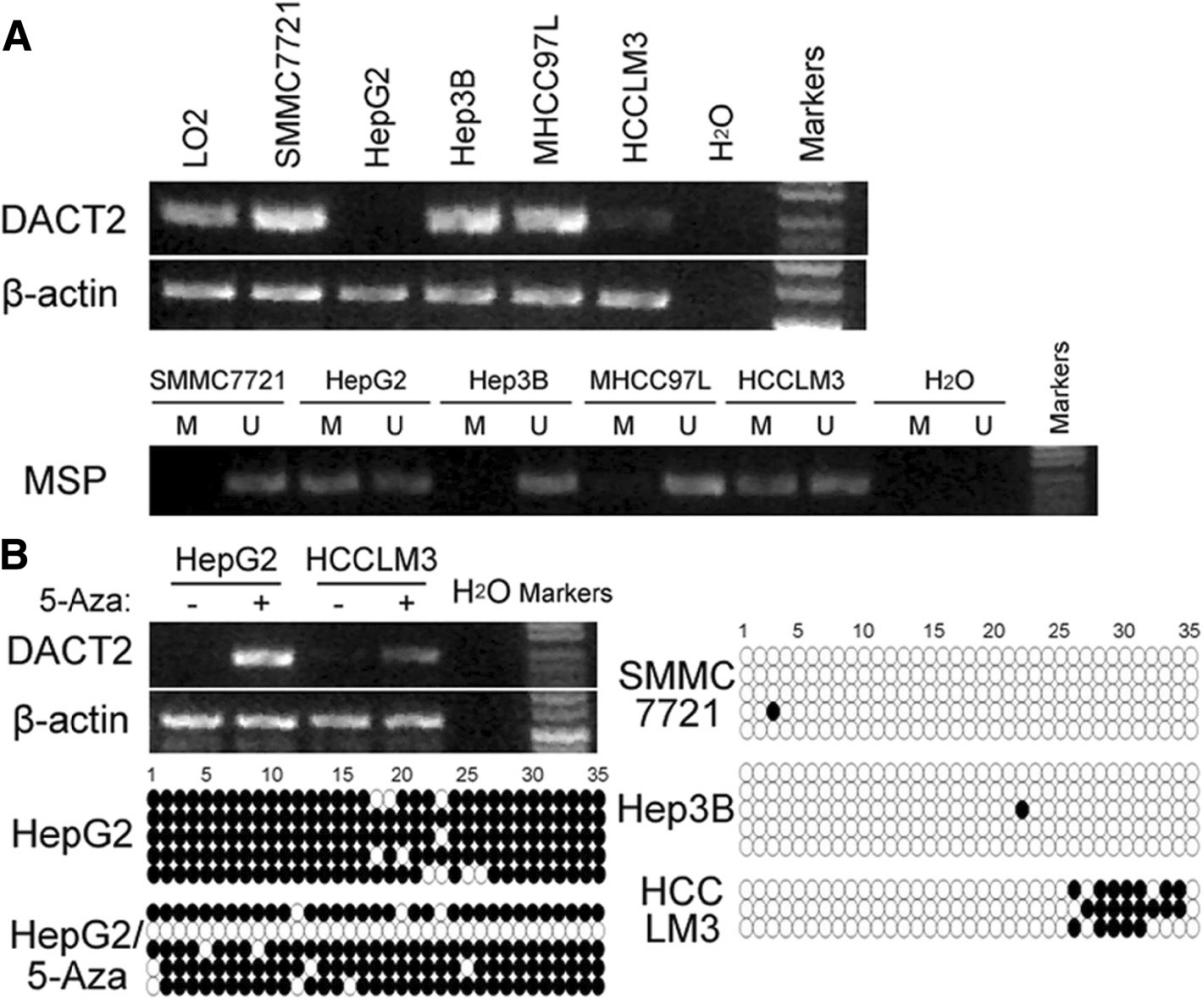
**(A) DACT2 mRNA expression and its methylation status in liver cancer cell lines.** (**B**) The mRNA expression of DACT2 was restored after treatment with 5-Aza. Demethylation was observed in the HepG2 cell line after treatment with 5-Aza, and the methylation of DACT2 in several liver cancer cell lines was examined by bisulfite genomic sequencing.

We first detected the expression level of DACT2 in five liver cancer cell lines. The results showed that DACT2 transcript was silenced or reduced in HepG2 and HCCLM3 cells (Figure 2A). To examine the role of promoter methylation in the silencing of DACT2, we further analyzed the methylation status of the DACT2 gene; the results indicate that the DACT2 gene promoter was partially methylated in cell lines with reduced or silenced expression (Figure 2A). To demonstrate whether methylation mediates DACT2 silencing, we treated two methylated cell lines that showed silencing of DACT2 with 5-Aza. 5-Aza restored DACT2 expression in both cell lines with obvious demethylation observed in the treated HepG2 cell line by bisulfite genomic sequencing (Figure 2B), suggesting that DNA methylation plays a crucial role in the transcriptional silencing of DACT2 in liver cancer cells.

### Cell cycle alterations after DACT2 silencing

**Figure 3:**
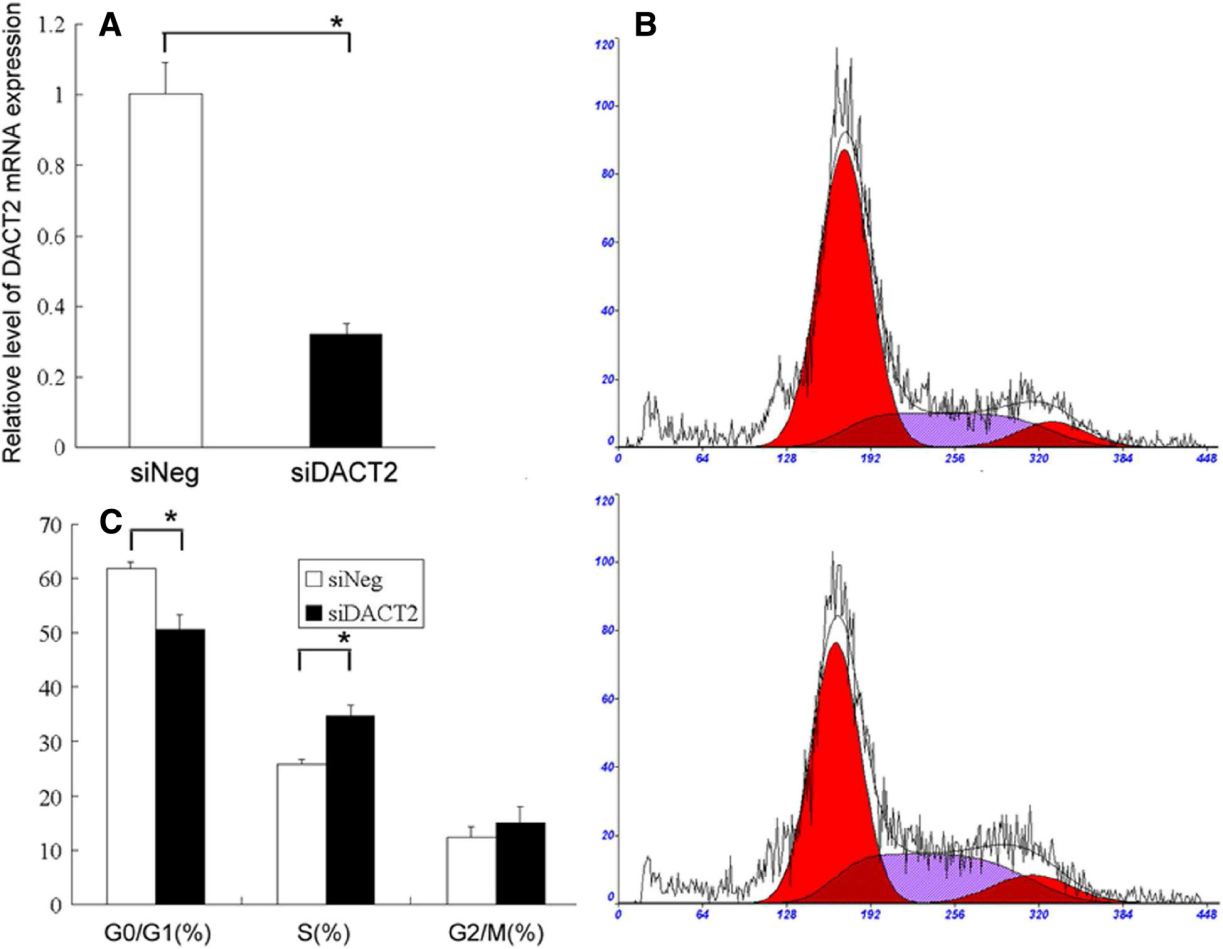
**Effect of DACT2 knockdown on cell cycle distribution of HCC cell line.** (**A**) Real-time PCR analysis showing expression of DACT2 in MHCC97L cell was significantly reduced after treatment with DACT2 siRNA compared with negative siRNA (*P < 0.01, Student’s *t-*test). (**B**) Cell cycle distribution of MHCC97L cell treated with DACT2 siRNA (*lower*) or negative control (*upper*). (**C**) The alteration of cell cycle distribution was different significantly (*P < 0.01, Student’s *t* test). All assays were performed in triplicate wells.

As shown in Figure 3A, the inhibitory efficiency of siRNAs for gene transcription was significant in morderate-metastasic potential MHCC97L cells. To determine the role of DACT2 in tumor cell cycle progression and tumor cell proliferation, we examined the effect of DACT2 knockdown in MHCC97L cells on the cell cycle. After 48 h of transfection, cell proliferation as determined by the number of cells in the S phase was increased from 25.9% to 34.6% (*P* < 0.01), as is shown in Figure 3B,C. Moreover, the number of cells in the G0/G1 phase decreased from 61.7% to 50.5% (*P* < 0.01).

### DACT2 silencing promotes cell migration and invasion

**Figure 4:**
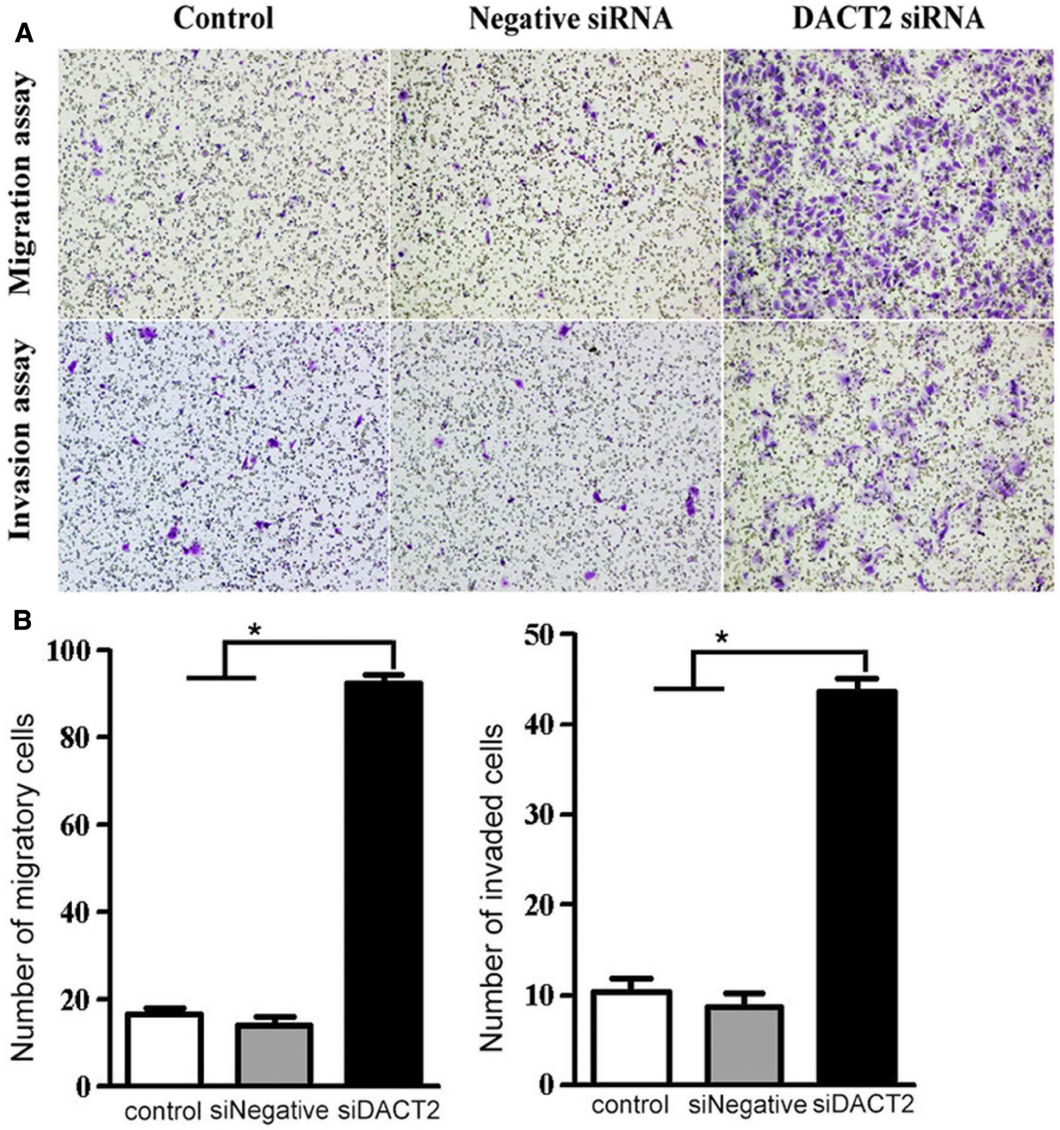
**Alteration of DACT2 expression in MHCC97L cell changes its migration and invasiveness *in vitro*.** (**A**) Representative images of migration and invasiveness of MHCC97L cell transfected with negative siRNA or siRNA against DACT2 (original magnification, ×10). (**B**) Selective knockdown of DACT2 led to a significant increase of migratory and invasive ability in MHCC97L (*P < 0.01, Student’s *t-*test). The columns indicate the number of migrated or invaded cells per field (original magnification, ×20).

The effect of DACT2 on the migration and invasiveness of HCC cells was analyzed using the Matrigel model. We found that the average number of migratory and invaded cells transfected with DACT2 siRNA was significantly increased when compared to those with negative control siRNA (Figure 4), indicating that the invasive potential of MHCC97L cells increased after DACT2 knockdown. This suggests that DACT2 also influences the migration and invasiveness of HCC cells.

## Discussion

In the current study, we found that the expression level of DACT2 was downregulated in human hepatocellular carcinoma by quantitative RT-PCR and immunohistochemical analysis. Similarly, in a previous study, Jiang et al. also found that DACT2 expression is also reduced in some colorectal tumors [19]. Therefore, DACT2 was implicated as a tumor suppressor gene in some types of human cancers. We further speculate about whether the expression level of DACT2 might be associated with clinicopathological parameters in liver transplantation HCC patients. Clinicopathological analysis revealed that HCC patients with low DACT2 expression was correlated with a larger tumor size (>5 cm) than those with high DACT2 expression. DACT2 may thus play a critical role in the proliferation and progression of HCC cells. To the best of our knowledge, there have been no prior reports studying the associations between DACT2 gene expression and clinicopathological parameters in human cancer. Thus, this is the first study to determine the expression pattern of DACT2 and report the clinical significance of DACT2 gene expression in HCC. Because of the possible role of DACT2 in tumor suppression and its reduced expression in cancer, the correlation of DACT2 expression and its methylation status have been characterized in colorectal cancer. Aberrant promoter hypermethylation of DACT2 was detected in several tumor samples with reduced DACT2 expression [19]. This finding led us to investigate the potential correlation of expression level and promoter methylation of DACT2 in HCC. Similar to the previous report on colon cancer cells, we found that DACT2 expression was present at very low or undetectable levels in some HCC cell lines and that 5-Aza-dC restored the transcription level of DACT2 (Figure 3). These data suggested that the downregulation of DACT2 expression was closely associated with promoter hypermethylation *in vitro*. Taken together, these findings further support that DACT2 is inactivated epigenetically in a number of human solid tumors including HCC and that promoter hypermethylation may be a critical mechanism for the transcriptional silencing of the DACT2 gene in liver cancer cell lines.

The tumor-suppressive function of DACT2 in HCC was investigated further by *in vitro* assays. In DACT2 expression silenced MHCC97L cells, FACS analysis revealed a significant increase of S phase cells and a decrease of G0/G1 phase cells, indicating G1/S arrest of the cell cycle and a significant promotion of cell proliferation. In addition, DACT2 knockdown in MHCC97L cells also resulted in increased ability of tumor cell invasion and metastasis (Figure 4). Taken together, these results suggest the role of DACT2 as a functional tumor suppressor gene through suppressing tumor cell proliferation, migration and invasion in HCC.

Our studies are the first to clearly identified the biological functions of DACT2 in human HCC cells; however, the molecular basis for how DACT2 knockdown leads to increased proliferation and invasion in HCC is still unknown. A number of studies have tried to illuminate the molecular mechanisms of the Dapper family proteins in regulating cell behavior. Waxman et al. found that zebrafish Dpr2 is a vital regulator of the noncanonical Wnt/Ca2+−PCP pathway [23]; however, Su et al. suggested that mouse Dpr2 inhibits the Wnt/JNK pathway and functions as a negative regulator of the TGF-β/Nodal signal pathway [24]. Thus, the Dpr2 protein may function to regulate distinct cell signaling pathways in a context-dependent manner. Therefore, the molecular mechanisms by which DACT2 exerts the tumor-suppressor effect in HCC needs further study.

It should be noted that there were some limitations in the current study. HCC is a tumor of diverse etiology, and our study population predominantly consisted of patients with hepatitis B-induced HCC. It is therefore important to determine whether DACT2 still functions as a tumor suppressor gene in patients with other underlying liver diseases. In addition, the study was confined to the Han Chinese population, which should be taken into account when evaluating the potential applicability of our findings to other ethnic populations. Lastly, the sample size was small because of the strict eligibility of the study population. The study should therefore be viewed as hypothesis generating, and our findings should be confirmed by examining larger clinical samples.

## Conclusion

We demonstrate that expression of DACT2 is downregulated in HCC compared to adjacent healthy liver tissues and that reduced DACT2 expression is significantly correlated with large tumor size. Furthermore, promoter hypermethylation is the principal regulatory mechanism of DACT2 inactivation in HCC cells. Functionally, DACT2 is an important tumor suppressor gene with key roles of regulating tumor cell proliferation, migration and invasion in the development and progression of HCC. Our study suggests that DACT2, which was silenced by promoter hypermethylation, may serve as a novel candidate tumor suppressor gene in HCC.

## Competing interests

The authors declare that they have no competing interests.

## Authors’ contributions

SSZ and LZ co-conceived the study and SSZ led the study. LZ, ZY and SG planned and executed experiments and analyses, supervised data acquisition, performed bioinformatic analyses, and carried out the experiments. LMW and JY provided pathological validation and technical support. ZYZ, FZ and LMW collected clinical samples. ZY, HYX and SG provided valuable input regarding study design, data analysis, and interpretation of results. ZY and SG wrote the manuscript. All authors read and approved the final manuscript.
